# Altered Central Autonomic Network in Baseball Players: A Resting-state fMRI Study

**DOI:** 10.1038/s41598-018-36329-9

**Published:** 2019-01-14

**Authors:** Jia-Hong Sie, Yin-Hua Chen, Chih-Yen Chang, Nai-Shing Yen, Woei-Chyn Chu, Yuo-Hsien Shiau

**Affiliations:** 1 Department of Biomedical Engineering, National Yang-Ming University, Taipei, 11221 Taiwan, ROC; 2 Research Center for Mind, Brain and Learning, National Chengchi University, Taipei, 11605 Taiwan, ROC; 3 Department of Psychology, National Chengchi University, Taipei, 11605 Taiwan, ROC; 4 Graduate Institute of Applied Physics, National Chengchi University, Taipei, 11605 Taiwan, ROC

## Abstract

The physiological adaptive regulation of healthy population with a high fitness level is associated with enhanced cognitive control in brain. This study further investigated the effects of different levels of sporting experience on intrinsic brain networks involved in central autonomic processing using resting-state functional magnetic resonance imaging. We explored functional connectivity of four core regions within central autonomic network (CAN), namely posterior midcingulate cortex (pMCC), left amygdala (AMYG), and right anterior (aINS) and left posterior insular cortices, in advanced and intermediate baseball players, and compared their strength of connectivity with individuals without baseball-playing experience. Functional connectivity maps across three groups confirmed a close relationship between CAN and large-scale brain networks in sensory, motor and cognitive domains. Crucially, both advanced and intermediate batters demonstrated enhanced connectivity between pMCC and sensorimotor network, between right aINS and dorsal anterior cingulate cortex, and between left AMYG and right putamen, than controls. These results reflected a stronger interregional coupling in sensorimotor and cognitive control, and in motor skill consolidation. In conclusion, we provided evidence that different levels of sporting experience could reorganize/enhance intrinsic functional connectivity for central autonomic processing.

## Introduction

The autonomic nervous system controls the functions of the internal viscera, blood vessels throughout the body, effectors in the skin and glands and all organs, except for voluntarily controlled striated muscles^1^. It interacts with perceptions, cognitions and emotions. For example, mental processes can influence autonomic responses to change physical state of the body as in respiration and skeletomotor activity, which might be intentional or incidental consequences of behavioral decision making^2^. The internal physiological state of the body can also influence mental processes to alter attention allocation or involvement of cognitive resources, for instance, arousal can enhance memory encoding^2^. Numerous studies have found that the long-term physical activity and exercise training have a great impact on autonomic control (i.e. improvement in blood volume expansion, cardiac remodeling, insulin resistance, and renal-adrenal function)^3,4^. Accumulating evidence in healthy population with a high fitness level, but not in athletes, also suggests that physiological adaptive regulation is associated with enhanced cognitive control in the brain^4,5^. Here we further asked whether the altered autonomic control due to sporting experience could extend to intrinsic brain networks.

Thanks to the advent of noninvasive brain imaging methods, recent studies have reported several areas that are consistently active for central autonomic processing during cognitive, affective and motor tasks; such areas include mid/dorsal anterior cingulate cortex (ACC), ventral medial prefrontal cortex, insula and amygdala (AMYG)^6,7^. A meta-analysis further identified four regions independently of task modality and referred to as the cores of the central autonomic network (CAN); these regions include posterior midcingulate cortex (pMCC), left AMYG, and right anterior (aINS) and left posterior insular cortices (pINS)^8^. The pMCC, also called cingulate motor regions, is located in the vicinity of the supplementary motor area (SMA) and mediates context-driven modulation of cardiac function via sympathetic output^9^. The AMYG works in tandem with cortical motor-related areas (e.g. SMA, primary motor cortex and premotor cortex) to facilitate or influence the perception of adaptive responses to emotional stimuli^10,11^ and has a strong linkage to sympathetic regulation. Interestingly, the left AMYG is also involved in parasympathetic regulation, possibly reflecting the need for balancing increased sympathetic and decreased parasympathetic outflow to aversive emotional stimuli^8^. The INS comprises two major functional subdivisions, namely, the right aINS that is related to the limbic system and the left pINS that is related to sensorimotor integration^12–14^. Similar to the left AMYG, the right aINS shows a dual role in autonomic function, that is, dichotomically with the ventral part associated with sympathetic regulation and with dorsal part associated with parasympathetic regulation^8^. The left pINS generally engages sympathetic modulation, for example, by showing anti-correlation with heart rate variability (a high frequency index) during movements^15^. Hence, the pMCC and the left pINS are involved in sympathetic regulatory function, and the left AMYG and the right aINS are involved in sympathetic and parasympathetic regulatory functions. These findings support the neurovisceral integration model^16–18^ for interoceptive awareness; this model has been widely used to explain the autonomic interaction with perceptions and emotions corresponding to the state of body^19,20^. Beissner and colleagues associated the divergent CAN subregions with distributed large-scale brain networks, particularly for task-positive and task-negative networks^8^. In particular, attentional and task-positive networks, which are connected with the aforementioned four seeds, show sympathetic predominance; whereas, affective and task-negative networks, which are related to the left AMYG and the right aINS, show a predilection toward parasympathetic regulation^8^. Moreover, task-positive and task-negative networks often fluctuate in the form of a negative correlation; their anti-correlation relationship is associated with cognitive functions, such as intra-individual variability in response time during Eriksen flanker task^21–24^. For instance, at greater anti-correlation between networks, the behavioural performance is less variable^24^.

Large-scale brain network models derived from resting-state functional resonance imaging (rs-fMRI) have been widely used to detect the plasticity of brain functions in the sensory, motor and cognitive domains^25,26^. The rs-fMRI is a technique that assesses the resting-state functional connectivity (rsFC) between distinct brain regions with the corresponding spontaneous fluctuation in the blood oxygen level-dependent (BOLD) signal when an individual is not performing an explicit task. Examination of this physiological coupling has been considered an effective method for studying the brain connectivity as shaped by sporting experience. For example, endurance athletes showed enhanced rsFC between the fronto-parietal network and distributed frontal regions, which are often associated with working memory and other executive functions^27^. Interestingly, they showed significant anti-correlations between the default-mode network (DMN; a classically reported task-negative network) and paracentral lobule (PCL) as well as postcentral gyrus (POST) which are greatly implicated in sensory and motor functions^27^. By contrast, world-class gymnasts showed decreased intra-network connectivity within the basal ganglia network, anterior DMN and fronto-parietal network^28,29^, as well as decreased inter-network connectivity between sensorimotor network (SMN) and basal ganglia network, and between posterior DMN and right fronto-parietal network, as compared to the controls^28^. The authors interpreted these results as evidence of an efficient coupling within and between networks developed in gymnastics training^28^. The aforementioned previous studies did not report consistent results possibly due to the specificity of different sports disciplines. However, in a general perspective, long-term motor skill training has been found to associate with neuroplastic adaptations in large-scale brain networks. Therefore, we were motivated to compare the effect of different levels of sporting experience on CAN related to large-scale brain networks. In this work, we recruited baseball players with different skill levels, that is, advanced and intermediate players, and compared their rsFC within CAN with individuals without any baseball-playing experience.

Baseball is a fast-ball sport and batting has been considered one of the most difficult motor skills. To hit a ball is a task with extremely severe spatiotemporal constraints^30^. Batters use the advance cue of the observed pitch and the ball trajectory to anticipate their action^31–33^. The abilities of quick stimulus discrimination, decision making and specific attention are developed by long-term extensive trainings, which are often started in very early childhood^34^. The involvement of attention and automatization of motor skills is associated with neuroplasticity in the brain^35,36^. Several distributed interconnected brain regions, such as precentral gyrus (PRE), POST, SMA and putamen, are associated with the acquisition, consolidation, and long-term retention of motor skills^36^. A recent meta-analysis reported alterations in brain activity of associative/premotor network and SMN, which are related to motor skill acquisition^37^, perceptual function and motor skill learning^38^. Notably, offline consolidation occurring after the end of a practice session may play an important role in behavioural skill improvement even without intervening practice^36,39^. This concept is compatible with the hypothesis of DMN linked to memory consolidation^40,41^.

Using rs-fMRI, this study aimed to investigate the effect of sporting experience on the rsFC of CAN seeded from pMCC, left AMYG, right aINS and left pINS, respectively. To more subtly tackle this issue, we recruited advanced batters (AB) and intermediate batters (IB) and compared each of their seed-based connectivity patterns with healthy controls (HC). In fact, in literature, several studies have investigated the rsFC associated to sensory, motor and cognitive functions^27–29,42–44^. However, no study has investigated rsFC in athletes that is particularly related to central autonomic processing. Therefore, we could not form a specific hypothesis but speculate that different levels of sporting experience would alter the rsFC of CAN depending on the characteristics of seed (i.e. sympathetic or parasympathetic regulation; attentional/task-positive or affective/task-negative network). In general, the strength of the four seed-based connectivities would increase or decrease (for positive and negative connectivities, respectively) as a function of baseball-playing experience as they represent sympathetic response function and principally engage in the attentional or task-positive network. Whereas left AMYG and right aINS additionally show parasympathetic regulation and involve affective and task-negative networks, especially within posterior DMN, the group differences might be shown in different patterns.

## Results

### Demographic characteristics of participants

As reported in Table 1, age significantly differed among the three groups. AB participants were younger than IB participants (*p* < 0.001, Bonferroni correction) and HC participants (*p* < 0.001, Bonferroni correction). To regress the effect of age difference, we considered age as a covariate of no interest in the imaging data analysis, as suggested in previous studies^45,46^. No significant differences in body mass index (*p* = 0.172) and Edinburgh handedness inventory (*p* = 0.347) were found among the three groups. Crucially, AB participants began to play baseball earlier than IB participants (*p* < 0.001) and had more baseball-playing experience in terms of years (*p* < 0.001) and weekly training hours (*p* < 0.001).

**Table 1:** Demographic and baseball-playing experience of participants.

Variable	AB	IB	HC	ANOVA or t test	*p*-value of post-hoc comparisons		
	N = 18	N = 15	N = 15	*p*-value	AB vs IB	AB vs HC	IB vs HC
Age (years)	20.6 ± 1.7	23.3 ± 1.3	23.0 ± 1.9	<0.001	<0.001^a^	<0.001^a^	1.000^a^
BMI (kg/m^2^)	24.8 ± 2.5	22.7 ± 3.5	23.8 ± 3.3	0.172	0.187^a^	1.000^a^	1.000^a^
EHI	81.8 ± 18.6	89.3 ± 15.4	88.0 ± 12.0	0.347	0.544^a^	1.000^a^	0.803^a^
AOC (years)	10.8 ± 2.7	19.1 ± 2.3		<0.001			
BE (years)	9.8 ± 2.7	4.2 ± 1.8		<0.001			
WT (hours)	19.7 ± 4.5	6.6 ± 2.3		<0.001			

Data presented as mean ± SD. AB, advanced batters; IB, intermediate batters; HC, healthy controls; N: sample size; ANOVA, analysis of variance; BMI: body mass index; EHI: Edinburgh handedness inventory; AOC, age of commencement; BE, baseball-playing experience; WT, weekly training. ^a^*p* value was obtained by post-hoc comparison with Bonferroni correction.

### Positive and negative seed-based functional connectivity maps across the three groups

**Figure 1:**
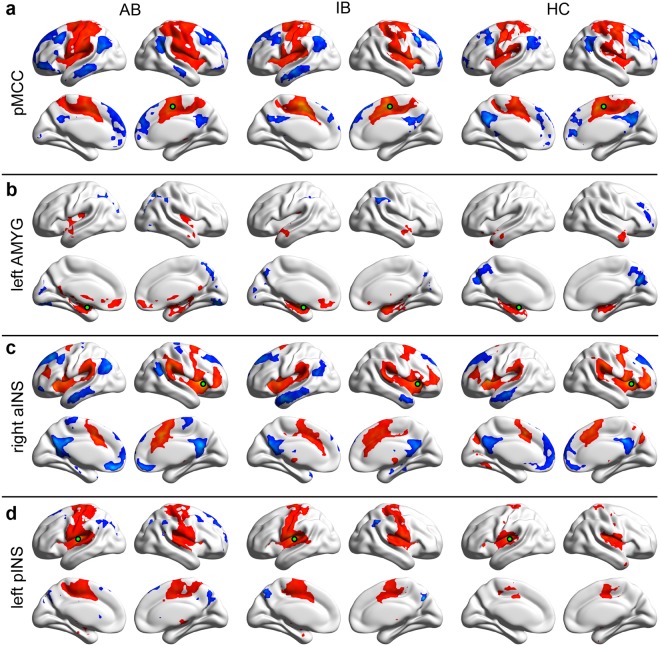
Positive and negative functional connectivity maps seeded from pMCC (**a**), left AMYG (**b**), right aINS (**c**) and left pINS (**d**) for advanced batters (AB), intermediate batters (IB) and healthy controls (HC) (pMCC, posterior midcingulate cortex; AMYG, amygdala; aINS, anterior insular cortex; pINS, posterior insular cortex; each seed was a 6 mm radius sphere shown in green; red and blue colour indicate positive and negative connectivity maps, respectively, with an AlphaSim correction threshold of *p* < 0.01).

As shown in the pMCC(+) maps of the three groups (Fig. 1a, red colour), pMCC was positively correlated with medial MCC and SMA, as well as INS, PRE, POST, supramarginal gyrus (SMG), inferior parietal lobule (IPL), superior frontal gyrus and superior parietal lobule, bilaterally. The negative correlations were apparent in the posterior cingulate cortex, precuneus, medial prefrontal cortex, lateral middle frontal gyrus, angular gyrus and middle temporal gyrus (Fig. 1a, blue colour). The left pINS was also positively correlated with pMCC, and its maps showed a large overlap with the pMCC(+) maps according to visual inspection (Fig. 1d, red colour). The negative maps of the three groups did not overlap (Fig. 1d, blue colour).

As shown in the left AMYG(+) maps of the three groups (Fig. 1b, red colour), the left AMYG was positively correlated with the limbic areas, including the right AMYG, ventral medial prefrontal cortex, bilateral hippocampus, parahippocampal gyrus. By contrast, negative correlations were apparent in the precuneus bilaterally (Fig. 1b, blue colour). The right aINS(+) maps of the three groups showed that the right aINS was positively correlated with the dorsal ACC extending to SMA, dorsolateral prefrontal cortex and inferior frontal gyrus, as well as SMG (Fig. 1c, red colour). Negative correlations were found in medial regions including ventral medial prefrontal cortex, posterior cingulate cortex and precuneus. Bilateral superior and middle frontal gyri and left angular gyrus and middle temporal gyrus were also involved (Fig. 1c, blue colour).

### Group differences in the positive and negative seed-based rsFC

**Figure 2:**
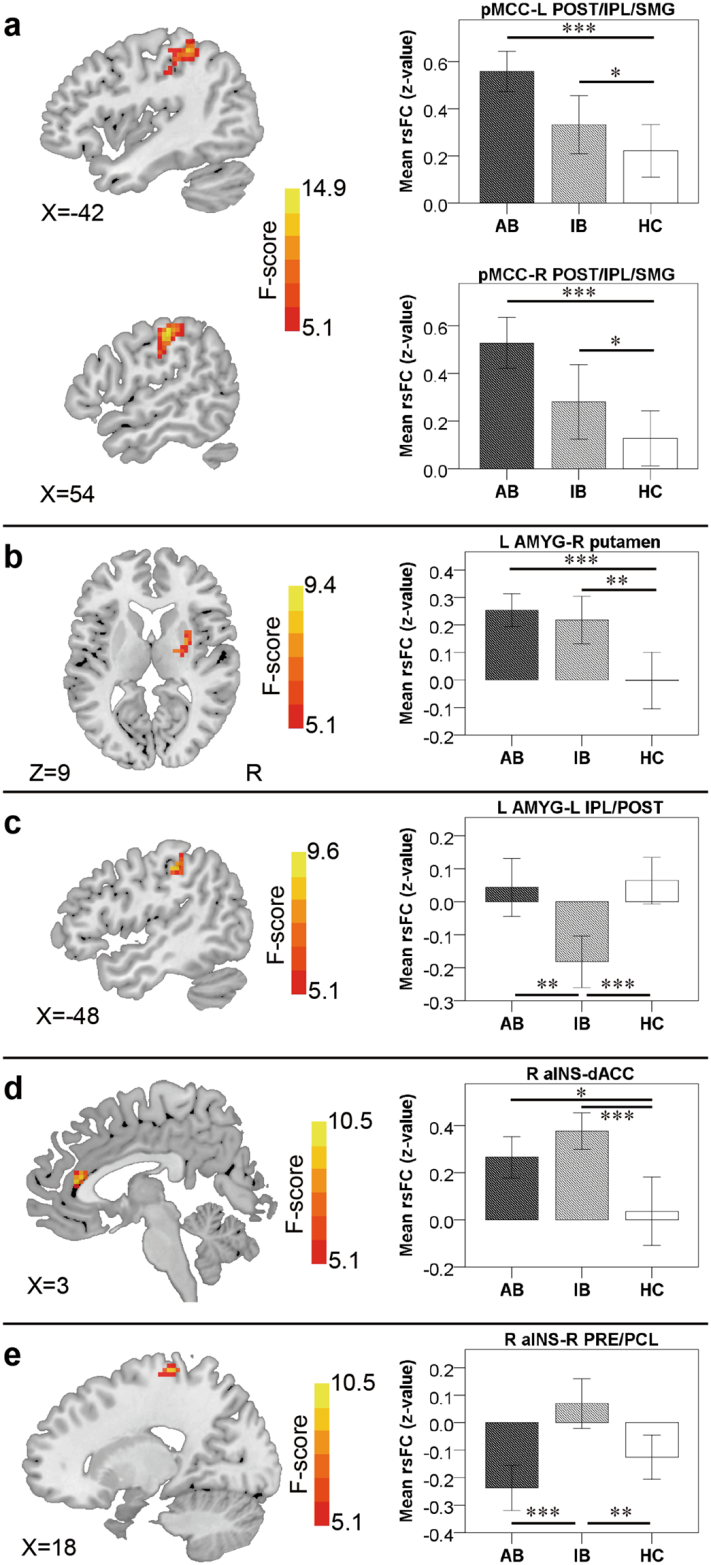
Regions showing group differences identified through ANCOVA (with an AlphaSim correction threshold of *p* < 0.01) and post-hoc comparisons in the pMCC(+) (**a**), left AMYG(+) (**b**), left AMYG(−) (**c**), right aINS(+) (**d**) and right aINS(−) networks (**e**), each with the corresponding mean strength between the seed and region for advanced batters (AB), intermediate batters (IB) and healthy controls (HC) shown in the bar plot, error bars indicate two standard errors, and asterisks indicate significant differences with Bonferroni correction (*p < 0.05, **p < 0.01, ***p < 0.001; pMCC, posterior midcingulate cortex; AMYG, amygdala; aINS, anterior insular cortex; pINS, posterior insular cortex; POST, postcentral gyrus; IPL, inferior parietal lobule; SMG, supramarginal gyrus; dACC, dorsal anterior cingulate cortex; PRE, precentral gyrus; PCL, paracentral lobule; L, left; R, right).

For the pMCC(+) networks, the ANCOVA detected a significant group effect in the bilateral POST extending to IPL and SMG. The strength of this rsFC increased as a function of baseball-playing experience (i.e. AB > IB > HC; Fig. 2a and Table 2); Indeed, post-hoc analyses confirmed that both AB and IB participants showed stronger rsFC than HC participants (*p* < 0.001 and *p* < 0.05, respectively). No group differences were found for the pMCC(−) networks. The left pINS(+) maps of the three groups were similar to those the pMCC(+) maps, but there were no group differences. With a more liberal threshold (a voxelwise *p* < 0.05 with an AlphaSim correction threshold of *p* < 0.05), the group differences were detected in similar regions as the pMCC(+) networks but a bit forward and medial, with one cluster in the left PRE extending to POST and the other one in the bilateral SMA. Similarly, the strength of these rsFC increased as a function of baseball-playing experience.

**Table 2:** Main regions showing significant differences in the pMCC(+), left AMYG(+), left AMYG(−), right aINS(+) and right aINS(−) networks among advanced batters (AB), intermediate batters (IB) and healthy controls (HC) by ANCOVA.

Region (BA)		MNI Coordinates			Peak F score	Cluster size
		x	y	z		(voxels)
pMCC(+): POST/IPL/SMG (2/40)	L	−42	−39	54	12.40	231
pMCC(+): POST/IPL/SMG (2/40)	R	54	−24	45	14.86	172
L AMYG(+): putamen	R	27	−3	9	9.38	27
L AMYG(−): IPL/POST (40)	L	−48	−30	45	9.61	37
R aINS(+): dACC (24)	B	3	33	15	10.45	40
R aINS(−): PRE (4)	R	18	−30	69	10.49	40

The voxel- and cluster-wise thresholds were set at p < 0.01, AlphaSim corrected. BA, brodmann area. MNI, Montreal neurological institute. pMCC, posterior midcingulate cortex; AMYG, amygdala; aINS, anterior insular cortex; POST, postcentral gyrus; IPL, inferior parietal lobule; SMG, supramarginal gyrus; dACC, dorsal anterior cingulate cortex; PRE, precentral gyrus; L, left; R, right; B, bilateral.

For the left AMYG(+) networks, the significant group effect was found in the right putamen. The strength of this rsFC also increased as a function of baseball-playing experience (i.e. AB > IB > HC; Fig. 2b and Table 2); post-hoc analyses indicated significant differences between AB and HC participants (*p* < 0.001) as well as IB and HC participants (*p* < 0.01). For the left AMYG(−) networks, the significant group effect was found in the left IPL extending to the POST, with IB participants showing the strongest connectivity among the three groups (i.e., IB < AB < HC in case of negative values; Fig. 2c and Table 2); the post-hoc analyses confirmed the significant differences between IB and HC participants (*p* < 0.001) as well as between IB and AB participants (*p* < 0.01).

For the right aINS(+) networks, the ANCOVA detected a significant group effect in the dorsal ACC where IB participants showed the strongest connectivity, followed by AB participants and HC participants showed the weakest connectivity (IB > AB > HC; Fig. 2d and Table 2). Post-hoc analyses indicated that significant differences between IB and HC participants (*p* < 0.001) as well as between AB and HC participants (*p* < 0.05). Finally, for the right aINS(−) networks, the ANCOVA detected a significant group effect in the right PRE extending to the PCL. AB participants showed the strongest connectivity, followed by HC participants and IB participants showed the weakest connectivity (AB < HC < IB in case of negative values; Fig. 2e and Table 2); the post-hoc analyses indicated the significant difference between AB and IB participants (*p* < 0.001) as well as between IB and HC participants (*p* < 0.05).

## Discussion

To investigate the potential effect of sporting experience in the central autonomic processing on the neural level, we examined the functional connectivity pattern involving CAN in individuals with very little, intermediate, and extensive baseball-playing experience (i.e. HC, IB and AB). The four core regions of CAN, namely pMCC, left AMYG, right aINS and left pINS, were used as seeds to track the positive and negative functional connectivity maps for the three groups, respectively. Our data provided empirical evidence that different levels of sporting experience selectively influenced the aforementioned seed-based functional connectivities that are specifically related to central autonomic processing; importantly, while the connecting areas were within sensorimotor areas, the strength of the positive connectivities generally increased as a function as sporting experience.

Firstly, regardless of little or extensive baseball-playing experience, pMCC and left pINS, which are the two core regions with sympathetic characteristic for the three groups, showed significant correlations with each other, and their connectivity maps were largely overlapped. In general, the positive functional connectivity of these two seeds were within the SMN comprising PRE, POST and SMA as well as IPL, which were functionally located in the primary and secondary motor and somatosensory regions. These results are consistent with previous findings as found in the healthy normal population^12–14^. Moreover, the strength of the connectivity of both pMCC and left pINS (with a more liberal statistical threshold) associating with the bilateral SMN, specifically in POST, IPL and SMG, depended on baseball-playing experience. That is, the greater the experience, the stronger the connectivity is. This result implied that baseball-playing experience might enhance the communication between the pMCC and left pINS with the SMN in processing multiple sensory inputs as well as in planning, initiating, executing or even inhibiting movements as required in baseball batting. Interestingly, IPL and SMG are typically considered as part of mirror neuron system^47–49^. Therefore, the stronger connectivity to IPL and SMG in batters might also reflect their tight coupling between the perception and execution of the batting movement as they had physically practised and perceived it extensively.

Secondly, for all participants, the left AMYG was positively correlated with the bilateral hippocampus, parahippocampal gyri and ventral medial prefrontal cortex, which are important components of limbic network. Such connections resemble those found in normal population^46,50–52^, implying the role of left AMYG in emotional and central autonomic processing through interactions with subcortical regions and prefrontal cortex^6,7^ as proposed by the neurovisceral integration model^16–18^. In addition, the strength of the connectivity between the left AMYG and the right putamen increased as a function of accumulated baseball-playing experience. The putamen is one of the main components of the basal ganglia network and plays a critical role in the planning, learning and execution of a new motor skill^35–37^. It is also involved in the cortico-basal ganglia loop when a newly learned motor skill became stable and consolidated in the retention phase^35^. Specifically, the right putamen is associated with motor skill acquisition at comparatively longer time scale (e.g. one month) in contrast to the aforementioned SMN at the short and medium time scales for immediate sensorimotor control^36,37^. Furthermore, the centromedial and superficial subvision of AMYG has been found to be positively correlated with striatum (e.g. putamen, caudate and globus pallidus), thalamus and ACC, bilaterally^52^, indicating the role of AMYG in facilitating the motor response, reward processing, attention and cortical readiness^52^. Taken together, the stronger coactivation between the left AMYG and right putamen found in AB and IB participants than in HC participants might reflect batters’ enhanced control of bodily arousal and affective processing, specifically for the motor skills that are consolidated or have become highly automated.

On the other hand, we found that the left AMYG was negatively correlated with the precuneus across three groups. This result is generally consistent with the previous findings of negative correlations with the bilateral precuneus, IPL, POST and PCL^46,50–53^. In fact, the mechanism underlying negative correlations has not yet been fully understood. Therefore, it has been suggested to find the correspondence between negative correlations and anatomy as a biological basis^54^. The AMYG is anatomically connected to precuneus in animals^55,56^ and to motor related areas (e.g., motor and premotor cortices)^10^ in humans, thus providing the biological basis to our result. Moreover, negative correlations have been also contributed to the use of global signal regression (GSR) even though the issue of GSR is still under debate^54^. Technically, regressing out the global signal has been found to alter the functional connectivity results^57,58^. However, importantly, Macey and colleagues have demonstrated dynamic changes of global signal during autonomic challenges, which reserves a unique variable independent of neural activation^59,60^. Alternatively, in our analysis, the global signal is regarded as a regressor to reduce low-frequency respiratory volume and cardiac effects^54,61^ as the three groups might potentially differ in the respiratory and cardiac function. Removing the global signal regressor in our data indeed weakened the significant differences of negative correlations found in the left AMYG (and also in the right aINS networks as shown in Supplemental Information). This result indicated that the left AMYG (as well as the right aINS) is more susceptible to the respiratory and cardiovascular fluctuations at rest as it is resided in the vicinity of the middle cerebral artery and vein. Future studies combining with structural connectivity would provide complementary evidence to avoid the limitation on the BOLD signal of fMRI. Interestingly, we found a group effect between the left AMYG and the left IPL extending to POST, with the significant difference only in the IB as compared to the HC and AB participants. As IB participants recruited in our study only played baseball for recreational purpose and did not reach the expert level, we suspected that this connection might be sensitive only in the early stage of expertise development. Future studies with a longitudinal design will help to confirm our speculation.

Thirdly, we found that the right aINS was positively correlated with task-positive networks across the three groups: one was the central executive network as anchored in dorsolateral prefrontal cortex and SMG, and the other one was the salience network as anchored in the ACC. Alternatively, the coactivation with dorsolateral prefrontal cortex and SMG could be considered the ventral fronto-parietal network^12–14^. Moreover, the right aINS was negatively correlated with DMN, including the posterior cingulate cortex, precuneus, angular gyrus and medial prefrontal cortex^12,62,63^. Given that INS is a functionally heterogeneous region, a network model of insula function has been postulated^22,23^. According to this model, the right aINS mediates information flow across the large-scale brain networks between task-positive and task-negative networks; the components of these networks flexibly interact with one another during the attention-demanding cognitive tasks and even in task-free states^21–24,62,63^. Our findings are in agreement with this model. Furthermore, the positive correlation between the right aINS and the dorsal ACC was stronger in the AB and IB as compared to HC participants. The dorsal ACC has been shown to frequently engage in pre-response conflict and decision uncertainty^64,65^, for example, when detecting conflict in the flanker task^66^. Currently, there is a wealth of evidence that the aINS and ACC have a unique functional relationship; they are considered together as input and output regions of interoceptive awareness that is engaged across cognitive, affective, and behavioral contexts^2,19,20,67^. Therefore, the enhanced positive connectivity in batters may reflect their refined interoceptive awareness that it is generally of fundamental importance across contexts, and specifically their better top-down control such as response selection or inhibition as required in baseball batting.

Finally, there was also a significant group effect in the strength of anti-correlations of the right aINS with PRE extending to PCL, with AB showing the strongest connectivity, followed by HC and then IB participants. The PRE is the site of primary motor cortex; and the PCL is located in the medial wall of SMN. A recent structural connectivity study also provided the evidence of the connection between the INS and primary motor and the somatosensory cortices^68^, providing the biological basis of our result. The enhanced anti-correlation specifically found in expert players as compared to intermediate players might reflect their particular functional associations to mediate communication in the sensory and motor circuits as required in high-level baseball playing scenarios. Even though HC participants showed the tendency of weaker connectivity than AB participants, they were not statistically different from AB participants. Moreover, they showed stronger connectivity than IB participants. At this moment, it seemed that the results could not be fully attributed to different levels of baseball-playing experience. Probably this connectivity is also sensitive to some other experience (rather than in sports), future studies are needed to solve this puzzle.

In conclusion, although state-of-the-art CAN studies mostly focused on cardiac autonomic balance regarding sympathetic and parasympathetic regulation of the heart, the present work used resting-state fMRI data to explore the patterns of rsFC driven by the core regions of CAN. With a cross-sectional design, we were able to subtly examine potential differences in the rsFC of CAN corresponding to different levels of sporting experience in baseball. The induced functional connectivity maps across the three groups confirmed a close relationship between CAN and large-scale brain networks in sensory, motor and cognitive domains: 1) both pMCC and left pINS were positively correlated with SMN. 2) left AMYG was positively correlated with limbic network, and negatively correlated with precuneus network. 3) right aINS was positively correlated with central executive network and salience network, and negatively correlated with DMN. Crucially, both AB and IB participants demonstrated enhanced positive connectivity of pMCC greatly associated with bilateral SMN, of right aINS associated with dorsal ACC, and of left AMYG associated with right putamen, as compared to individuals without any baseball-playing experience; and the strength of the connectivity generally increased as a function of baseball-playing experience. These results reflected a stronger interregional coupling in the sensorimotor and cognitive control as well as motor skill consolidation due to baseball-playing experience. Our findings provided empirical evidence that intrinsic functional connectivity for central autonomic processing could be reorganised and enhanced thanks to different levels of sporting experience.

## Methods

### Participants

Fifty-two healthy young male adults aged 18–25 years were recruited to participate in the study. All participants were healthy and free of psychiatric history or neurological illness. The participants included 19 AB, 15 IB and 18 HC. AB participants were recruited from highly ranked Taiwanese university baseball teams competing in the first division for several years. IB participants were recruited from department baseball teams of different universities as recreational level group. The HC participants did not have any baseball-playing experience or regular physical exercise habit. All of the participants did not have specific experience in other sports. All participants completed the Edinburgh Handedness Inventory to ensure right hand dominance^69^. Before the experiment, all of the participants gave written informed consent to the study. All methods were performed in compliance with the ethical principles of the Declaration of Helsinki and were approved by the Research Ethics Committee of National Taiwan University.

### Data acquisition and image preprocessing

Participants received instructions to rest with their eyes open while the sign ‘+’ centrally projected in white against a black background. Images were collected on a Siemens Skyra 3.0 T MRI scanner (Erlangen, Germany). Resting-state fMRI scans were collected using an echo-planar imaging sequence with a repetition time (TR) of 2000 ms, an echo time (TE) of 25 ms, a 90° flip angle (FA), and a 3 mm slice thickness. The acquisition matrix was 64 × 64, with a 216 mm × 216 mm field of view (FOV). Each scan session was 360 s long and comprised 180 functional volumes, with each volume consisting of 41 axial slices. For spatial normalisation and localisation, T1-weighted images were acquired using the following magnetisation-prepared rapid gradient echo (MPRAGE) sequence: TR/TE = 2530/3.3 ms, inversion time (TI) = 1100 ms, FA = 7°, FOV = 256 mm × 256 mm, voxel size = 1.0 mm × 1.0 mm × 1.0 mm, number of slices = 192.

Image preprocessing was carried out using Data Processing Assistant for rs-fMRI (DPARSF)^70^ in Data Processing and Analysis for Brain Imaging toolbox (DPABI)^71^, implemented in Statistical Parametric Mapping (SPM8) (http://www.fil.ion.ucl.ac.uk/spm). The preprocessing included the following steps: (1) the first 10 volumes of each participant were discarded, (2) slice timing correction for timing offsets using sinc interpolation and (3) head motion correction using a six-parameter spatial transformation. One AB participant and three HC participants were excluded under the criterion with head motion more than 2.0 mm or 2.0° of head rotation. To limit nuisance covariates from head movement, global signal, white matter and cerebrospinal fluid^61,62^, (4) the functional data were then processed by using multiple regression analysis: (*i*) head motion signals from step 3, (*ii*) white matter and cerebrospinal fluid by setting a probability threshold 0.99 on one’s own tissue segmentation maps based on his structural image and (*iii*) global signal within a group mask generated by including voxels present in at least 90% of all participants. The GSR was used because it helps remove non-neuronal confounds such as respiration and cardiac activity^54,61^. More importantly, it could improve the specificity of positive correlations and the correspondence to anatomical connectivity^54^. Next, the individual structural images were then (5) co-registered to the resulting functional data for each participant and (6) subsequently spatially normalised to the Montreal Neurological Institute (MNI) space by using the unified segmentation and resampled to 3 mm isotropic voxels and (7) a Gaussian kernel of 4 mm full width at half maximum for spatial smoothing. Finally, (8) the temporal band-pass filtering (0.01–0.1 Hz) was carried to reduce low-frequency drift and high-frequency physiological aliasing.

The peak of the four seeds in CAN were defined following the results of Beissner, *et al*.^8^, including pMCC (MNI coordinates: 4, 0, 48) and left pINS (−32, −18, 12) with sympathetic characteristics, and right aINS (34, 20, 4) and left AMYG (−22, −8, −16) with both sympathetic and parasympathetic characteristics. Each seed was a sphere with 6 mm radius. The time course of each seed was correlated with time course of each voxel (within 90% coverage of group mask) for each participant. The strength of rsFC was defined as Fisher transformed correlation coefficients (i.e. z values) for improving the normality.

### Statistical analysis

The individual functional connectivity maps for each group underwent two-tailed one-sample t test (compared with zero) to determine significant positive and negative correlations with the seeds of pMCC, left AMYG, right aINS and left pINS voxel by voxel, respectively. We used AlphaSim correction (*p* < 0.01) based on the Monte Carlo simulation algorithm to correct for multiple comparisons, with a voxelwise *p* < 0.01 and cluster size at least > 244 within each network mask depending on 1000 simulations corrected for each group to include voxels present in 90% of participants. The surface visualisations were illustrated using BrainNet Viewer^72^. The union set of the resultant 3D significant positive or negative connectivity maps of all groups for each seed were used as masks for subsequent group-level analysis, referring to pMCC(+), pMCC(−), left AMYG(+), left AMYG(−), right aINS(+), right aINS(−), left pINS(+) and left pINS(−) network, respectively. Statistical analyses across the three groups for each network were conducted using one-way analysis of covariance (ANCOVA), with age as a covariate^45,46^. The same threshold adjustment method for group level analyses was used (i.e., a voxelwise *p* < 0.01 and cluster size at least > 23 depending on 1000 simulations corrected for each connectivity, which yielded an AlphaSim correction threshold of *p* < 0.01). Finally, the individual z values from the regions that showed significant group effect by ANCOVA were extracted and compared for all pair comparisons of the three groups with Bonferroni correction as post-hoc analyses using Statistical Package for the Social Sciences 20.0 (SPSS, Chicago, Ill, USA).

## Electronic supplementary material

Supplementary information
