# Supplementary material for: Altered Central Autonomic Network in Baseball Players: A Resting-state fMRI Study
Source: Sci Rep. 2019 Jan 14;9:110. doi: 10.1038/s41598-018-36329-9 (PMC6331574; doi:10.1038/s41598-018-36329-9)
Supplement: Supplementary file 1 — Supplementary information [file 41598_2018_36329_MOESM1_ESM.docx]

Altered Central Autonomic Network in Baseball Players: A Resting State fMRI Study

Jia-Hong Sie^1,+^, Yin-Hua Chen^2,+^, Chih-Yen Chang^2^, Nai-Shing Yen^2,3,*^, Woei-Chyn Chu^1,*^, Yuo-Hsien Shiau^2,4,*^

**Supplementary Information**

**Results**

**Group differences without the GSR**


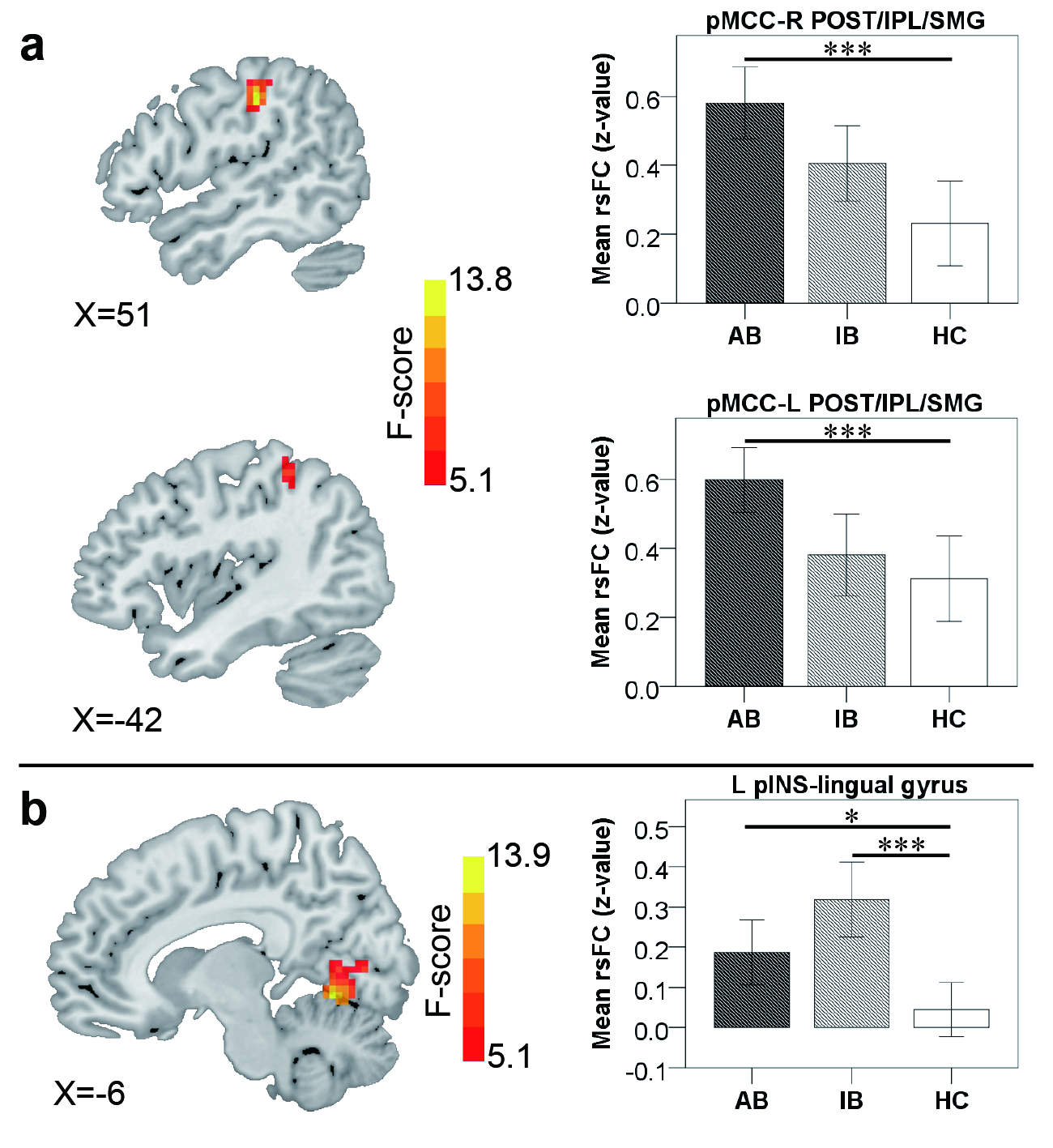


**Figure 1.** Regions showing group differences identified through ANCOVA (with *p* < 0.01, AlphaSim corrected) and post-hoc comparisons when we did not regress out global signals in the pMCC(+)(a) and left pINS(+) networks (b), each with the corresponding mean strength between the seed and region for advanced batters (AB), intermediate batters (IB) and healthy controls (HC) shown in the bar plot, error bars indicate two standard errors, and asterisks indicate significance with Bonferroni correction (* *p* < 0.05, *** *p* < 0.001; pMCC, posterior midcingulate cortex; pINS, posterior insular cortex; POST, postcentral gyrus; IPL, inferior parietal lobule; SMG, supramarginal gyrus; L, left; R, right).

**Table 1.** Main regions showing significant differences in the pMCC(+) and pINS.L(+) networks among advanced batters (AB), intermediate batters (IB) and healthy controls (HC) by ANCOVA without global signals.

|  |  | **MNI Coordinates** | | | **Peak F score** | **Cluster size** | |
| --- | --- | --- | --- | --- | --- | --- | --- |
| **Region (BA)** |  | **x** | **y** | **z** | **or T value** | **(voxels)** |  |
| pMCC(+): POST/IPL/SMG (40) | R | 51 | −24 | 42 | 13.75 | 105 |  |
| pMCC(+): POST/IPL/SMG (40) | L | −42 | −39 | 54 | 7.93 | 64 |  |
| L pINS(+): lingual/calcarine | B | -6 | −51 | −3 | 13.9 | 251 |  |

Thresholds were set at *p* < 0.01, AlphaSim corrected. BA, brodmann area. MNI, Montreal neurological institute. POST, postcentral gyrus; IPL, inferior parietal lobule; SMG, supramarginal gyrus; L, left; R, right; B, bilateral.
